# Pediatric Cholestatic Liver Disease: Review of Bile Acid Metabolism and Discussion of Current and Emerging Therapies

**DOI:** 10.3389/fmed.2020.00149

**Published:** 2020-05-05

**Authors:** Alyssa Kriegermeier, Richard Green

**Affiliations:** ^1^Division of Gastroenterology, Hepatology and Nutrition, Department of Pediatrics, Feinberg School of Medicine, Ann & Robert H. Lurie Children's Hospital of Chicago, Northwestern University, Chicago, IL, United States; ^2^Division of Gastroenterology and Hepatology, Department of Internal Medicine, Feinberg School of Medicine, Northwestern University, Chicago, IL, United States

**Keywords:** pediatric, cholestasis, bile acid, bile acid receptor, treatments

## Abstract

Cholestatic liver diseases are a significant cause of morbidity and mortality and the leading indication for pediatric liver transplant. These include diseases such as biliary atresia, Alagille syndrome, progressive intrahepatic cholestasis entities, ductal plate abnormalities including Caroli syndrome and congenital hepatic fibrosis, primary sclerosing cholangitis, bile acid synthesis defects, and certain metabolic disease. Medical management of these patients typically includes supportive care for complications of chronic cholestasis including malnutrition, pruritus, and portal hypertension. However, there are limited effective interventions to prevent progressive liver damage in these diseases, leaving clinicians to ultimately rely on liver transplantation in many cases. Agents such as ursodeoxycholic acid, bile acid sequestrants, and rifampicin have been mainstays of treatment for years with the understanding that they may decrease or alter the composition of the bile acid pool, though clinical response to these medications is frequently insufficient and their effects on disease progression remain limited. Recently, animal and human studies have identified potential new therapeutic targets which may disrupt the enterohepatic circulation of bile acids, alter the expression of bile acid transporters or decrease the production of bile acids. In this article, we will review bile formation, bile acid signaling, and the relevance for current and newer therapies for pediatric cholestasis. We will also highlight further areas of potential targets for medical intervention for pediatric cholestatic liver diseases.

## Introduction

Causes of chronic liver injury in children include a wide variety of congenital and acquired diseases. The primary cholestatic diseases of infancy and childhood are frequently symptomatic and often rapidly progressive. These chronic cholestatic diseases that present in childhood include biliary atresia (the most common cause of cholestatic liver disease in children), Alagille syndrome, progressive familial intrahepatic cholestasis diseases (PFIC), bile acid synthesis defects, cystic fibrosis related liver disease, ductal plate abnormalities including Caroli syndrome and congenital hepatic fibrosis, primary sclerosing cholangitis (PSC), and certain metabolic diseases.

Many patients ultimately require liver transplantation and within the pediatric population, cholestatic liver diseases remains the leading indication for pediatric liver transplant in the U.S, accounting for nearly half of all pediatric patients listed for liver transplant (1). For this article, we will review the formation and secretion of bile and bile acid signaling with a highlight on disease causing mutations, and review current and emerging treatments that may potentially decrease cholestatic injury and alter disease progression within pediatric cholestatic liver diseases. A comprehensive review of supportive management for cholestatic patients such as management of nutritional deficiencies and symptomatic management of pruritus is beyond the scope of this manuscript, though these are important to patient outcomes.

## Cholestasis

Cholestasis describes an impairment in bile flow resulting in the accumulation of the components of bile (bile acids, bilirubin, cholesterol, and phospholipids) which often leads to clinical symptoms and serum laboratory abnormalities. The production of bile is influenced greatly by the enterohepatic circulation and recycling of bile acids. The impediment to bile flow in cholestasis may be due to obstructive causes (e.g., biliary atresia, obstructive choledochal cysts, common bile duct obstruction) or secondary to any impairment of appropriate synthesis or secretion of the components of bile from the hepatocytes and cholangiocytes. The formation of bile and its flow into the intestinal lumen serves both as an excretory function of the liver within its role to metabolize and detoxify substances, and as an aid in digestion of fat and fat-soluble vitamins. When bile acids are retained within cells, their detergent nature and induced signaling pathways can lead to significant cell damage. The exact mechanism of injury in human and animal models of cholestasis is likely multifactorial (i.e., altered inflammatory responses, increased fibrinogenesis, induction of apoptosis or autophagy) and may be different depending on the precise nature of the injury, the setting of injury (*in vivo* vs. *in vitro*) and the species incurring the injury; and these detailed mechanisms of cholestatic injury have been reviewed in detail elsewhere (2). Since bile acid synthesis and transport occurs primarily within the hepatocytes and cholangiocytes, with potentially high intracellular concentrations of bile acids, the liver is the primary site of damage in settings of cholestasis.

## Bile Synthesis

Cholesterol is the precursor for the synthesis of bile acids. Within the hepatocyte, cholesterol is converted into the primary bile acids cholic acid (CA) and chenodeoxycholic acid (CDCA) in humans by a complex biochemical pathway involving a number of different hepatic enzymes within the “neutral” (classic) pathway or the “acidic” bile acid synthesis pathways. In the initial and rate limiting step within the neutral pathway, cholesterol is modified with the addition of a hydroxyl group at the C-7 position by the microsomal cytochrome P450 liver-specific enzyme cholesterol 7α-hydroxylase (CYP7A1) (3, 4). In the acidic pathway, which contributes significantly to the production of chenodeoxycholic acid, a mitochondrial cytochrome P450 enzyme (sterol 27-hydroxylase, CYP27A1), leads to an initial side-chain oxidation of cholesterol (5). After these initial steps within the neutral or acidic pathway, continued modifications occur culminating in the end products of cholic acid or chenodeoxycholic acid. In the neutral pathway, 12α-hydroxylation of products (by a liver specific microsomal cytochrome P450 12α-hydroxylase, CYP8b1) directs intermediates to the production of cholic acid, and therefore is important in determining the ratio of the production of cholic acid to chenodeoxycholic acid (6). Interestingly, the ratio of cholic acid to chenodeoxycholic acid varies during human development. Specifically, fetal bile has a predominance of chenodeoxycholic acid with a ratio of cholic:chenodeoxycholic acid of reportedly ~0.85 suggesting altered activity of the 12a-hydroxylase enzyme, or alternative pathways of bile acid production during fetal life (7). Neonates and adults have a predominance of cholic acid but notably different ratios of cholic:chenodeoxycholic acid of ~2.5 and 1.6, respectively (7, 8).

After primary bile acids are produced, they are conjugated to either glycine or taurine largely within peroxisomes (9). Several congenital deficiencies in enzymes involved in the pathways of bile acid synthesis have been described, broadly termed bile acid synthesis defects (BASD). BASD include diseases that are characterized by single enzyme defects (SED) in bile acid synthesis pathway proteins, or diseases of peroxisome formation, such as Zellweger spectrum disorders which can lead to the accumulation of toxic bile acid intermediates and may present as significant cholestasis in the newborn period (10). The newborn infant has a predominance of taurine conjugated bile acids, whereas older infants and adults have a predominance of glycine conjugated bile acids (8, 11, 12). Additionally, significant species variation exists with regards to specific bile acid biosynthesis, metabolism, and the proportion of glycine or taurine conjugation of primary bile acids which is important when evaluating effects of potential bile acid altering drugs in animal models. For example, humans produce primarily CA and CDCA as described above, however mice predominantly produce muricholic acids; and humans primarily form glycine conjugated bile acids (but have the ability to form taurine conjugates), however, mice form nearly exclusively taurine conjugates (12, 13). These differences leads to a far less hydrophobic bile acid pool in mice (14). The reader is referred to the excellent reviews of bile acid metabolism in different species for more in depth review (2, 15).

Once conjugated, bile acids must be secreted into the canalicular lumen to become a component of the bile that will ultimately be excreted into the intestinal lumen. Bile acids are secreted across the canalicular membrane via an ATP cassette transporter known as the bile salt export pump (BSEP) which is encoded by the gene *ABCB11*. Mutations in *ABCB11* lead to the disease PFIC type 2 in humans (16). After being secreted, bile acids may undergo “cholehepatic” circulation, whereby bile acids may be reabsorbed back across the cholangiocyte border and transported back to hepatocytes or the portal circulation. This proposed mechanism of “cholehepatic shunting” is likely particularly relevant for specific bile acid derivatives including nor-ursodeoxycholic acid (17–20).

In addition to BSEP, there are several other specific transporters at the canalicular membrane that are responsible for excreting the other components of bile across this membrane. Phospholipids, primarily phosphatidylcholine (PC), are secreted via the multidrug resistance P-glycoprotein 3 in humans (MDR3, gene *ABCB4*) which is known as mdr2 in mice (21, 22). While homozygous mutations within *ABCB4* may lead to PFIC type 3, patients with a mild phenotype or who are heterozygous for mutations in *ABCB4* have been found in increasing numbers in several cholestatic conditions of adulthood including low phospholipid-associated cholelithiasis syndrome (LPAC) and intrahepatic cholestasis of pregnancy (ICP) (23, 24). PFIC type 1 disease in humans is caused by homozygous mutations with the FIC1 protein (encoded by the ATPase member *ATP8B1* gene) which is located at the hepatocyte canalicular membrane, and apical membrane of cholangiocytes and enterocytes (25). The exact mechanism resulting in cholestasis secondary to *ATP8B1* mutations remains unclear, however evidence suggests FIC1 is an aminophospholipid transporter which regulates inner and outer lipid content of the plasma membrane and if mutated may alter the canalicular membrane integrity; additionally FIC1 mutations may lead to alterations in the activity of the farnesoid X receptor (FXR), a nuclear receptor critical to bile acid homeostasis (26, 27). Additional mutations recently discovered in the tight junction protein 2 (TJP2, gene *TJP2*), also known as zona-occludens 2, can lead to progressive intrahepatic cholestasis and has been referred to as PFIC4, however as more newly discovered causes of inherited progressive cholestasis are discovered, naming of the intrahepatic cholestasis diseases based on the mutated gene rather than a numbering system initially developed at a time prior to identification of the responsible mutations is superior.

Cholesterol is secreted via the heterodimer transporter ABCG5/ABCG8 (genes *ABCG5/ABCG8*) also called sterolin, and mutations in *ABCG5/ABCG8* genes can cause sitosterolemia, which has a varied clinical presentation including associated liver disease (28, 29). Conjugated bilirubin and other glucoronidated molecules are secreted via the multidrug resistance-related protein 2 (MRP2, gene *ABCC2*), and mutations in *ABCC2* lead to Dubin-Johnson syndrome (30). Rotor syndrome is another disease characterized by a benign increase in conjugated bilirubin, caused by simultaneous mutations in two members of the OATP family (OATP1B1, gene *SLCO1A2* and OATP1B3, gene *SLCO1B3*) located on the hepatocyte sinusoidal membrane which serve to reabsorb conjugated bilirubin (31).

Other components of bile such as water, bicarbonate, chloride, and other electrolytes have an important role in bile homeostasis and are regulated within cholangiocytes (32). The membrane protein cystic fibrosis transmembrane conductance regulator (CFTR, gene *CFTR*), a chloride channel important for bicarbonate secretion into bile, is located on the apical membrane of cholangiocytes, and the chloride/bicarbonate exchanger AE2 (gene *SLC4A2*) is on the apical membrane of cholangiocytes and on the canalicular membrane surface (33). Mutations within *CFTR* lead to cystic fibrosis and cystic fibrosis related liver disease (CFRLD). AE2 knock-out mice develop a phenotype similar to the adult cholestatic liver disease primary biliary cholanigitis (PBC) (34). Bile flow and water composition is aided by water channels or aquaporins (AQP) within the cholangiocyte membranes as well (33).

Additionally, mutations leading to abnormalities in the normal development of the biliary system can lead to cholestasis as occurs in Alagille syndrome and ductal plate malformations. Alagille syndrome is an autosomal dominant, multisystem disorder that frequently involves the liver, classically characterized by bile duct paucity on pathology, and is caused by mutations in *Jagged1* (*JAG1*) or *Notch2*. Ductal plate malformations refer to cholangiopathies associated with the lack of normal development and remodeling of the intrahepatic bile ducts that occurs along the branches of the developing portal vein. These include entities such as Caroli syndrome and congenital hepatic fibrosis which are most commonly associated with autosomal recessive polycystic kidney disease (ARPKD), secondary to mutations in the gene *PKHD1* which encodes fibrocystin (35). A list of these discussed cholestatic diseases associated with bile transport and signaling can be found in Table 1.

**Table 1:** Cholestatic diseases associated with bile transport and signaling.

**Disease (inheritance)**	**Protein involved**	**Gene(s)**
PFIC 1 (AR)	FIC1	*ATP8B1*
PFIC 2 (AR)	BSEP	*ABCB11*
PFIC 3 (AR)	MDR3	*ABCB4*
PFIC disease due to TJP2 mutations (PFIC4) (AR)	TJP2	*TJP2*
PFIC disease due to FXR mutations (PFIC5) (AR)	FXR	*NR1H4*
Bile acid synthesis defects (BASD) (AR)	*varies (single enzymes, peroxisome proteins)	*varies based on disease
Sitosterolemia (AR)	Sterolin (ABCG5/ABCG8)	*ABCG5/G8*
Dubin-Johnson syndrome (AR)	MRP2	*ABCC2*
Rotor syndrome (AR)	OATP1B1/ OATP1B3	*SLCO1A2, SLCO1B3*
Cystic fibrosis related liver disease (AR)	CFTR	*CFTR*
Alagille syndrome (AD)	*Notch signaling pathway	*JAG1, NOTCH2*
Caroli syndrome/congenital hepatic fibrosis (associated with ARPKD) (AR)	fibrocystin	*PKHD1*

AR, autosomal recessive; AD, autosomal dominant. *Multiple.

## Enterohepatic Circulation

After the production and conjugation of primary bile acids within the hepatocytes, these compounds are then secreted along with the other components of bile into the intestine where they are ultimately metabolized by bacterial enzymes into secondary bile acids. Cholic acid and chenodeoxycholic acid are deconjugated and then may be dehydroxylated into deoxycholic (DCA) and lithocholic acid (LCA), respectively, which constitute the majority of bile acids excreted into the feces in humans (33, 36, 37). Ursodeoxycholic acid (UDCA) may also be produced from the epimerization of CDCA, but generally is found at low concentrations in humans (38, 39). While 3–5% of bile acids are excreted in the feces, the majority of primary and secondary bile acids are reabsorbed in the terminal ileum and return to the liver via the portal vein where they are again excreted via the process known as enterohepatic circulation (40). The hydrophobicity of bile acids affects their solubilization properties (detergent effects) and therefore their deleterious effects on cell membranes, cell signaling, as well as their influence on choleresis (41). The hydrophilic bile acid UDCA and its taurine conjugate, tauroursodeoxycholic acid (TUDCA), are weaker detergents and do not cause significant membrane/cellular toxicity whereas LCA is very hydrophobic and cytotoxic (42).

**Figure 1:**
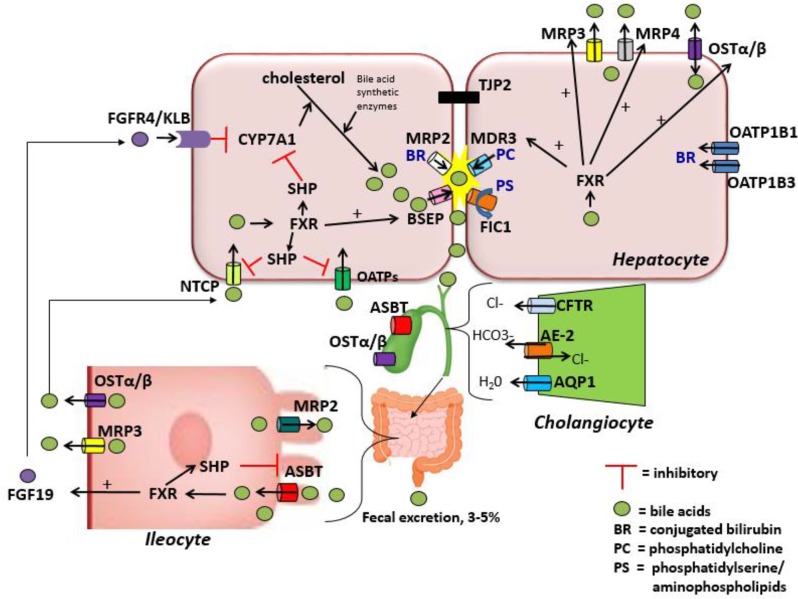
Enterohepatic circulation of the components of bile.

Bile acid uptake occurs at the enterocyte via the apical sodium-dependent bile salt transporter (ASBT) also known as the ileal bile acid transporter (IBAT) encoded by the gene *SLC10A2* (43, 44). ASBT is also found on the luminal membrane of large bile ducts and the gallbladder (33). ASBT transports conjugated bile salts into the enterocyte, which interact with ileal bile acid-binding protein (I-BABP) within the cytosol (45). Bile acids are then exported across the basolateral membrane via a heteromeric transporter, organic solute transporter alpha and beta (OSTα-OSTβ). In addition to the enterocytes of the terminal ileum, OSTα-OSTβ is located on the basolateral membrane of hepatocytes and cholangiocytes as well as several other tissues and can function to export bile acids from the hepatocyte back to the sinusoidal blood if necessary (33, 46). At the hepatocyte basolateral membrane, bile acids are then transported from the sinusoidal blood into the cell via the sodium-taurocholate cotransporting polypeptide (NTCP) primarily in humans, but also by members of the anion transporting polypeptide family (OATP) in mice (47, 48). They are then excreted once more into bile, thus completing the enterohepatic circuit (Figure 1). During cholestatic conditions, the hepatocyte basolateral membrane also has pumps that serve to efflux bile acids back into the sinusoidal blood including MRP3 (encoded by gene *ABCC3*) and MRP4 (encoded by gene *ABCC4*), in addition to OSTα-OSTβ as mentioned above (49). A in depth review of bile acid enterohepatic circulation with a significant focus on intestinal metabolism of bile acids can be found elsewhere (50).

## Bile Acid-Activated Receptors and Regulation of Bile Acid Synthesis and Flux

In addition to the greater understanding of the recirculation of bile acids, over the past 20 years there have been several receptors identified as bile acid activated receptors (BAR) that are activated by bile acids and that have significant effects on the regulation of bile acid synthesis and metabolism. Most notably, the discovery of the first BAR, farnesoid X receptor (FXR), has led to dramatic reshaping of the understanding of the tight regulation of this process. FXR (encoded by the *NR1H4* gene—nuclear receptor subfamily 1, group H, member 4) is a nuclear hormone receptor that influences bile acid synthesis by forming a heterodimer with retinoid X receptor (RXR) and binding directly to the regulatory elements (FXR-responsive elements, FXR-RE) of its target genes, as well as by regulating the downstream transcription factor small heterodimer partner (SHP) (51–54). Mutations in *NR1H4* may lead to another phenotype of progressive intrahepatic cholestasis in children that has been referenced as PFIC5 previously, but are better described as cholestasis secondary to *NR1H4* mutations (55).

After activation by bile acids, FXR regulates several aspects of bile acid trafficking and production. FXR induces the expression of SHP which binds another receptor, LRH-1 (liver receptor homolog 1), preventing it from interacting with promotor regions on both CYP7A1 and CYP8B1, therefore negatively regulating bile acid synthesis within the hepatocyte (56). FXR also regulates the release of fibroblast growth factor-19 (FGF-19) (FGF-15 in mice) from ileal enterocytes which travels via the portal circulation to the hepatocytes and binds the FGF receptor FGFR4 and β-Klotho (KLB), another transmembrane protein that functions as a co-receptor required for FGF-19 binding in the liver (57). Within the liver, FGF19 signaling suppresses bile acid synthesis by repressing CYP7A1 expression (58). In the presence of bile acid-mediated FXR activation in the ileum, SHP activation leads to decreased expression of ASBT, through repression of LRH-1 resulting in decreased bile acid update by the ileum (59).

FXR, through SHP, represses the expression of NTCP and OATP which consequently reduces the reuptake of bile acids from the portal circulation back into the hepatocytes (60). Additionally, FXR increases the transcription of the BSEP gene *ABCB11* (61, 62). The net effect of FXR activation is therefore to promote bile acid excretion and reduce bile acid update, helping to maintain homeostasis within the hepatocyte. On the hepatocyte basolateral side, FXR activation leads to increased expression of OSTα/β, MRP3, and MRP4 which in the setting of cholestasis can efflux bile salts from the basolateral surface of the hepatocyte to reduce intracellular bile acid concentrations and therefore mitigate the toxic effects of bile acids to the cell (46, 63).

In addition to FXR, there are several other bile-acid activated receptors including the liver X receptor (LXR), the pregnenolone X receptor (PXR; known as steroid and xenobiotic receptor or SXR in humans), the vitamin D receptor (VDR), and the constitutive androstane receptor (CAR). PXR is activated by LCA and likely serves to reduce bile acid toxicity via its regulation of CYP7a1 and other cytochrome p450 enzymes, OATP2, and MRP2 (64–66).

An alternative family of receptors known as the G-protein-coupled receptor family, the most notable of which is GPBAR1 also known as TGR5 (previously also referred to as M-BAR), are also able to bind bile acids (specifically secondary bile acids) (67–69). TGR5 (GPBAR1) has several cell-signaling and immunoregulatory effects within liver disease due to its presence on Kupffer cells and natural killer (NK) cells. Additionally, through its expression on sensory nerves, TGR5 (GPBAR1) may have a role in regulating pruritus (70–72).

The structure and conjugation of each bile acid affects its hydrophobicity which influences the ability to activate the different receptors discussed above. CDCA is the most potent naturally occurring ligand of FXR followed in strength by DCA = LCA > CA (69, 73, 74). Secondary bile acids (LCA and DCA) are potent activators TGR5 (GPBAR1) (68, 69).

In addition to bile acid receptor regulation of bile acid synthesis, other pathways regulating bile acid synthesis have been discovered recently. The unfolded protein response (UPR) is an adaptive cellular response pathway to endoplasmic reticulum (ER) stress that functions to regulate protein homeostasis, but can also trigger apoptosis. Activation of the UPR has been described in several liver diseases including fatty liver disease, viral hepatitis, and cholestasis (75, 76). However, more recently animal and *in vitro* data has shown that the UPR pathways function to reduce ER stress and hepatic injury as well as regulate bile acid synthesis and transport, and that activation of FXR in turn also influences the UPR (77). ER stress has been demonstrated to suppress CYP7A1-dependent production of bile acids independently of FXR regulated pathways, and to alter the expression of the bile acid transporters BSEP and MRP3 (78, 79).

## Medical Management of Pediatric Cholestasis

Historically, pediatric chronic cholestasis due to all causes has primarily been managed symptomatically. Pediatric patients with cholestatic liver diseases frequently suffer from significant symptoms and complications including poor growth secondary to fat malabsorption, fat soluble vitamin deficiencies, hepatic osteodystrophy, and complications related to progressive portal hypertension such as ascites and gastrointestinal bleeding. Significant chronic pruritus is common in several of the genetic syndromes and a significant cause of major morbidity. Agents such as ursodeoxycholic acid, bile acid sequestrants, and rifampicin have been the mainstays of treatment for years with the understanding that they may promote choleresis or alter the composition of the bile acid pool. However, patient response to these medications is frequently insufficient and data to show that they alter disease progression in most conditions remains lacking. Patients frequently require high calorie supplemental formulas to improve malnutrition secondary to fat malabsorption and antihistamines and other medications to aid in pruritus management and sleep. Fat soluble vitamin levels should be monitored closely and repleted if required, though deficiencies may be difficult to correct (80, 81). However, in light of the improved understanding of the intricate signaling pathways involved in BA metabolism, newer strategies are being developed and considered for treatment that hope to alter disease progression. It is also likely that with a more nuanced understanding of the underlying etiologies of patients with cholestasis, combination therapies tailored to specific patients may be employed. The following paragraphs and Table 2 will highlight the current, and potential future medical therapies for pediatric cholestatic liver diseases.

**Table 2:** Current and potential medical therapies in pediatric cholestatic liver diseases.

**Therapy**	**Proposed mechanisms of action**	Pediatric disease with a favorable outcome reported in animal models (*or human subjects)
UDCA	More favorable bile composition, increased expression of BA transporters, reduced apoptosis	*PSC at standard doses, *PFIC3
Nor-UDCA	Increased choleresis, cholehepatic shunting, and bicarbonate secretion	*PSC and PFIC3 (mdr2^−/−^ mouse), CFRLD, A1AT*
Cholic acid	Decrease synthesis of toxic bile acid intermediates	*BASD (approved for use in BASD 2015)
Rifampin	PXR agonist, altered gut flora	
Bile acid sequestrants	Increased fecal BA secretion; increased hydrophilic BA, decreased inflammation and fibrosis; increased biliary proliferation	*PSC and PFIC3 (mdr2^−/−^ mouse)*
Chemical chaperones	Improved trafficking of transport proteins to membrane surface	*PFIC1, *PFIC2, A1AT, CFRLD
ASBT inhibitors	Increased fecal BA secretion	PSC and PFIC3 (mdr2^−/−^ mouse), *PSC, *PBC, *ALGS
FXR and TGR5 agonists	Suppressed BA synthesis, increased BA secretion across canalicular membrane	PSC and PFIC3 (MDR2–/– mouse) *PBC
FGF19 analogs	Suppressed BA synthesis	*PSC
Anti-inflammatory/Anti-fibrotic therapies, hepatocyte or stem-cell transplants	CCR2/CCR5 inhibition, multiple anti-inflammatory/anti-fibrotic pathways	*Rat fibrosis model, PFIC3 (mdr2^−/−^ mouse), Biliary atresia (RRV mouse model)*

## Ursodeoxycholic Acid (UDCA)

Ursodeoxycholic acid, a hydrophilic bile acid, has been used for decades for intra-hepatic and extra-hepatic cholestatic diseases in childhood. Although originally approved for gallstone dissolution, it is considered first-line therapy for the adult disease primary biliary cholangitis (PBC) and has been shown to improve long term outcomes including reduction in the risk of death or need for liver transplant for PBC patients (82). However, strong data showing long term improvement in outcomes in pediatric chronic cholestatic diseases is lacking. By enriching the BA pool with hydrophilic UDCA, this treatment was originally thought to decrease the toxic effects that hydrophobic bile acids may exert on cells in the setting of cholestasis, however it has been proven that the size of the hydrophobic bile acid pool remains the same with UDCA treatment despite improvements in cholestasis (83). UDCA has been shown to promote choleresis, and alter the expression of the BA transporter BSEP and also MDR3 (84, 85). Part of the choleretic effect of UDCA may be secondary to increased chloride and bicarbonate secretion from the cholangiocytes (86, 87). UDCA may also function to reduce apoptosis via its beneficial effects on mitochondrial membrane stabilization (88, 89). Taurine conjugated UDCA (TUDCA) has been demonstrated to reduce ER stress in obese mice and may also impact the bile acid pool (90). Finally, UDCA may function to reduce immunoglobulin and cytokine production, making it potentially beneficial in decreasing inflammatory consequences of cholestasis (91, 92). Notably, much of this work has been performed *in vitro* and in rodent models and the full range of effects in human disease are still uncertain. In adults with PSC, there were several studies that showed improvement in biochemical markers on UDCA (on doses between 10 and 15 mg/kg/day), but failed to show improvement in long term outcomes (93–96). Additionally a trial using high dose UDCA (28–30 mg/kg/day) for the treatment of adult PSC lead to improved liver biochemical markers, but significantly increased the risk of serious events including portal hypertension, death and liver transplantation compared to placebo (97). The current AASLD guidelines for adults recommend against the use of UDCA in PSC as a medical therapy (98). However, pediatric patients with PSC are not the same population as adult patients and it is important to evaluate the use of UDCA in these patients separately. Retrospective studies in children with PSC have shown improved biochemical markers after treatment with more standard dosing of UDCA, and while no improvement in long term outcomes specific to UDCA has been found in any of these studies, patients who are treated with UDCA are more likely to have normalization of biochemical markers which is a predictor of improved long term outcomes (99–102). A recent pilot trial (NCT01088607) investigating withdrawal and then reinstitution of UDCA in pediatric PSC was completed in 2017, which showed approximately 1/3 of patients had no significant change in labs with withdrawal of UDCA, while 1/3 of patients met criteria for a disease flare with withdrawal of UDCA, though long term differences in these 2 subsets were not reported (103). This suggests that there may be a subset of patients who have biochemical evidence of UDCA response, and that those patients may have improved outcomes arguing for continuation of UDCA in these patients. For patients with PFIC, particularly PFIC3, UDCA may be effective at improving pruritus and laboratory values in a majority of patients, however there are no proven long term benefits on disease progression (104). A recent Cochrane review of the use of UDCA in cystic fibrosis related liver disease found that there was no evidence regarding its effects on long term outcomes, and no strong evidence of its effectiveness in this disease (105).

Despite this lack of strong evidence in favor of its use, given the low side effect risk profile of standard dose UDCA (10–20 mg/kg/day), it is often employed in cases of pediatric chronic cholestasis.

## 24-Norursodeoxycholic Acid (NorUDCA)

NorUDCA is similar to UDCA but has a shortened side chain and is a potent choleretic agent (18, 106). It strongly induces bicarbonate secretion into bile, and is relatively resistant to amidation (conjugation) (unlike UCDA which is conjugated to glycine or taurine). It decreases the amount of phospholipid and cholesterol secretion relative to bile acids in bile (18). Given that NorUDCA is resistant to amidation, it undergoes extensive “cholehepatic” circulation, the proposed process where bile acids are absorbed across the cholangiocyte canalicular boarder and transported back to the portal circulation and hepatocytes where they can be re-secreted. This likely contributes to the significant induction of bicarbonate secretion and cholangiocyte choleresis seen with NorUDCA (17). In several rodent models of liver disease, including the mdr2(^−/−^) mouse model of PSC and PFIC3, NorUDCA has been shown to be more effective than UDCA in preventing liver disease progression, and there is some evidence that NorUDCA may have anti-fibrotic effects (107, 108). In the *CFTR* knockout mice mouse model of cystic fibrosis, NorUDCA leads to increased biliary bicarbonate and fluid secretion (20). In a study using a mouse model of A1AT deficiency, NorUDCA lead to decreased accumulation of misfolded protein, and improvement in liver disease likely through increased autophagy mechanisms (109, 110). A study of NorUDCA in adult patients with PSC demonstrated significant reduction in alkaline phosphatase levels and an excellent safety profile, and there is a phase 3 clinical trial currently enrolling patients as young as 16 years of age (NCT03872921) (111). Given these features, NorUDCA may prove to have increased utility in several pediatric liver diseases including cystic-fibrosis related liver disease, A1AT deficiency, and PSC, however, future studies are needed.

## Cholic Acid

Bile acid synthesis disorders (BASD) are a group of rare metabolic diseases that are characterized by single enzyme defects (SED) in bile acid synthesis pathway proteins or Zellweger spectrum disorders (ZSDs). Oral cholic acid was approved for use in BASD in 2015 and has been shown to decrease synthesis of toxic bile acid intermediates, and improve histologic features on liver biopsy in certain patients with BASD (112, 113). Though some patients treated with cholic acid in an open label study continued to have disease progression, it is proposed that these patients had advanced liver disease prior to starting treatment and that for patients newly diagnosed with these rare diseases, if therapy is initiated promptly, disease progression can be halted and liver transplantation may be avoided (112). This is in contrast to UDCA which does not satisfactorily reduce atypical bile acid intermediates in BASD, and when given in combination therapy with cholic acid did not provide additional benefit and may have decreased cholic acid efficacy (113). Given these outcomes, cholic acid is one of the few effective medical therapies for a pediatric chronic cholestatic disease shown to alter disease progression and should be considered for BASD.

## Rifampicin

Rifampicin is an antibiotic frequently used off-label in pediatric and adult patients with cholestatic pruritus (114, 115). Rifampicin is a strong agonist of the nuclear receptor PXR, which induces hepatic transport proteins and metabolic enzymes including “detoxifying” cytochrome P450s. This may explain some of its efficacy in the pruritus associated with cholestasis, although the exact pruritogenic agents remain unclear. Activation of PXR may mitigate bile acid toxicity via its suppression of CYP7a1, and induction of OATP2 and MRP2 expression, and by increasing CYP3A enzyme activity which functions to further detoxify bile acids via hydroxylation and urinary excretion (85). Though some studies have actually shown increased CYP7a1 activity in humans with treatment of rifampicin (contrary to the *in vitro* data), these studies have also shown a decrease in secondary, hydrophobic bile acids (LCA and DCA). Therefore, alternative explanations for a reduction in pruritus may include alterations of intestinal flora which alters production of secondary bile acids, or additional excretion of other possibly pruritogenic compounds via increased expression of MRP2 or other liver canalicular membrane transporters (85, 116, 117). Rifampicin has been associated with drug-induced hepatitis, however, and therefore pediatric patients with cholestatic liver disease should be monitored routinely if this medication is prescribed (118). However, as it is generally well-tolerated it is still frequently employed for pruritus management in pediatric patients but additional studies may be helpful to elucidate any disease modifying benefits.

## Bile Acid Sequestrants

Cholestyramine and colesevelam are bile acid sequestrants that are also frequently used off label for the treatment of pediatric cholestatic pruritus but are also lacking strong data that they influence any long term outcomes related to chronic liver disease in pediatric patients (119). Although these drugs were initially developed for treatment of hypercholesterolemia, by binding bile acids they can enhance fecal bile acid secretion, and can be effective for some patients with pruritus (120). Notably, treatment with colesevelam significantly reduced inflammation and fibrosis in in an animal model of PSC and PFIC 3 (mdr2^−/−^ mice) through several proposed mechanisms including increased hydrophilic hepatic/biliary bile acids, and increasing colonic resin bound BA which are more abundant TGR5 ligands, which raises the possibility of specific benefits of bile acid sequestrants in PSC and PFIC3 outside of simply treating pruritus (121). This study also proposed a possible benefit of improved appropriate cholangiocyte proliferation in the setting of injury which may prevent progression to ductopenia, raising the possibility that it may potentially be beneficial in other ductopenic diseases including Alagille syndrome. Further well-controlled pediatric studies in specific cholestatic diseases are required. As bile acid sequestrants have been used off label for decades in pediatrics, they are generally considered safe but are not uncommonly associated with GI side effects including constipation, bloating, and abdominal discomfort. Additionally, their poor palatability often makes compliance an issue within the pediatric population and long term use of bile acid sequestrants can lead to fat malabsorption and fat soluble vitamin deficiencies so these need to be monitored in patients while on therapy.

## Chemical Chaperones and Endoplasmic Reticulum (ER) Stress Modulators

For pediatric cholestatic liver diseases secondary to genetic mutations that alter the functionality of the canalicular transport proteins, chemical chaperones that may aide in protein folding and increase functional protein delivery to membranes are potentially therapeutic. 4-phenyl butyrate (4-PBA), has been used for treatment of hyperammonemia secondary to urea cycle defects given its nitrogen scavenging properties. However, it is also a chemical chaperone that may bind to areas of misfolded protein, preventing aggregation, enhance proper protein folding, and therefore increasing delivery of such proteins to their target locations (122). 4-PBA has been shown to decrease markers of endoplasmic reticulum stress and reduce cell death in animal models of several diseases and as such its benefits may be multifactorial (123–125). Case reports in patients with PFIC 1 and 2 have suggested improvement in symptomatic pruritus after 4-PBA treatment and have even demonstrated increased trafficking of the BSEP and FIC1 protein to the canalicular membrane in other studies (126–130). 4-PBA in cell and mouse models of A1AT deficiency have demonstrated improved secretion of A1AT mutant protein from cells, however a preliminary small human study did not show increased serum levels of A1AT after 14 days of oral 4-PBA therapy (131, 132). Treatment also lead to side effects in several patients including nausea, vomiting and elevated uric acid levels. Chemical compounds developed to enhance CFTR folding may also improve trafficking of FIC1 to canalicular membrane surfaces and therefore may be a potential therapeutic option for multiple hepatic diseases (133). Rodent disease models of type 2 diabetes have shown that treatment with TUDCA reduced ER stress and likely improves protein folding capacity (90). It should be noted, that there are case reports of potential significant side effects of 4-PBA including severe hepatotoxicity and psychiatric disease (134, 135). Additionally, patients who have mutations other than missense mutations that lead to decreased protein trafficking may not benefit from these types of drugs. However, the prospect of chaperone and ER stress modulators is an area of potential new therapeutic targets and further studies are warranted.

## Apical Sodium Dependent Bile Acid Transporter (ASBT) Inhibition

Given its essential role in the enterohepatic circulation of bile acids, the ASBT is a potential therapeutic target for cholestatic diseases. Animal work has previously demonstrated that treatment with ASBT inhibitors reduced total bile acid composition, and improved liver chemistries and fibrosis in mdr2^−/−^ mice (136, 137). There have been several studies looking at the ASBT inhibitor maralixibat (LUM001) or linerixibat (GSK2330672) in adults with both PSC and PBC (NCT02061540, NCT01904058) (138). Diarrhea and other GI symptoms were commonly reported with these drugs, and while some studies showed improvements in itching, others lacked significant differences in itching scores. Several studies demonstrated improvement in bile acids, and one study demonstrated modest improvement in biochemical features of disease such as bilirubin and alkaline phosphatase, though this changes were not clearly clinically meaningful and there is no evidence that these ASBT inhibitors altered the long-term natural history of PBC or PSC in these trials.

A double-blind, placebo-controlled phase 2b study of pediatric patients with Alagille syndrome reported no significant differences in adverse events between maralixibat and placebo and no significant changes in serum bile acids or liver chemistries compared to patients who received placebo, which suggests this drug appears safe for use in pediatric patients (139). While this study failed to show a significant decrease in itch measurements between all drug doses and placebo, there was a significant decrease in the subset of patients taking the 2 lower doses of the drug (139). Notably, this study was only able to randomize 6 patients to the arm with the highest maralixibat dosing, and the limited patient size may have contributed to the negative results. Phase 2, 3, and long term safety/efficacy studies of the ASBT inhibitor maralixibat (LUM001) are ongoing at different centers internationally in pediatric patients with cholestatic liver diseases including but not limited to PFIC and Alagille syndrome (NCT02057718, NCT02047318, NCT02117713, NCT02160782, NCT04168385, NCT03905330, NCT04185363). Another ASBT inhibitor, odevixibat (A4250) is currently undergoing a phase 3 study in pediatric patients with PFIC type 1 and 2 (NCT03566238, NCT03659916).

Most of these studies are primarily evaluating improvement in clinical symptoms such as pruritus or improvement in serum laboratory values including serum bile acids, but they may help determine if patients also have some long term benefit such as decreased rate of transplant or increased survival with native liver.

## FXR and TGR5 (GPBAR1) Agonists

Given its extensive influence on the regulation of bile acids, FXR agonists have been developed for the treatment of cholestasis. The first synthetic FXR ligand GW4064 was extensively studied in animals, but had low bioavailability and was not pursued for human drug development. However, a semisynthetic derivative of CDCA (6-ethyl-CDCA), now commonly referred to as obeticholic acid (OCA), was developed and has an FXR agonist potency that is 100-fold greater than CDCA (140, 141). OCA has been studied in adults, primarily with primary biliary cholangitis (PBC) and has been shown to improve serum alkaline phosphatase levels in patients who did not tolerate or who did not have an adequate response to UDCA, but also increased pruritus in a high proportion of patients (142–144). In addition, OCA may be potentially beneficial in non-alcoholic steatohepatitis, although it can cause dyslipidemia and may worsened insulin resistance. In adults, OCA is now approved for patients with PBC with an inadequate response or intolerance to UDCA, but is also being studied in adult patients with non-alcoholic steatohepatitis (NASH), PSC and other liver disorders.

Additionally, a second generation FXR agonist (INT-767) has been developed that is reported to be a 3-fold more potent FXR ligand than OCA and is also a TGR5 (GPBAR1) ligand. In the mdr2(^−/−^) mouse model of PSC and PFIC3, INT-767 treatment lead to improved biliary fibrosis and hepatic inflammation as well as induced bicarbonate rich bile production which was superior to improvements seen in selective FXR and TGR5 (GPBAR1) treatments alone (145). Given the presence of TGR5 (GPBAR1) on Kuppfer and immune cells as described above, this may be an additional pathway by which bile acid receptor ligands may serve to mitigate the effects of chronic cholestasis.

Trials of additional non-bile acid FXR modulators (cilofexor and tropifexor) are ongoing in adults with PBC, PSC, and NASH and preliminary human trials indicate that they may have potential therapeutic benefits with less side effects including pruritus (146–148). Given these promising studies in adults, particularly those that show improved fibrosis markers, future trials in pediatric cholestatic liver diseases involving FXR and TGR5 agonists and other bile acid receptor ligands may be considered.

## FGF19 Analogs

FXR activation promotes the release of ileal FGF19 (the human homolog of murine FGF15) which suppresses bile acid synthesis by repressing CYP7A1 expression and therefore FGF19 analogs may be a potential therapeutic target for pediatric cholestatic liver diseases. However, there are concerns that FGF19 over-expression in mice lead to hepatocellular carcinoma, and concerns about a potential risk of the development of cholangiocarcinoma in cholestatic biliary diseases (149). However, an FGF19 analog which reportedly is not tumorigenic has been developed prompting increased interest for human use (150). This purportedly non-tumorigenic FGF19 analog (NGM282) has been evaluated in adult PSC patients and reduced serum BA levels as well as ALT/AST values, but did not decrease alkaline phosphatase levels (151). Promisingly, there were also decreased serum biomarkers of hepatic fibrosis, however the study may not have been of sufficient duration to demonstrate if there were any long term improvements in these patients. Studies are ongoing with this treatment in patients with PBC and also NASH and potential use in children may be considered pending new safety and efficacy data. It is important to note that FXR agonists can also induce endogenous FGF19 production. In addition, since carcinogenetic stimuli may have a long latency period prior to tumor formation, concerns regarding any tumorigenic potential of FGF19 may require long-term safety studies in order to more definitively demonstrate its safety.

## Anti-Inflammatory and Antifibrotic Agents

Given the often rapidly progressive nature of fibrosis and fibro-inflammatory liver damage in many pediatric cholestatic diseases, agents that specifically reduce inflammation and hepatic fibrosis are desirable. Unfortunately, the limited number of clinical trials that have attempted to modulate fibro-inflammatory responses in biliary atresia, specifically with corticosteroids and intravenous immunoglobulin, have not demonstrated any improvement in outcomes (152, 153). In adults, there are active clinical studies looking at modulation of several fibrosis signaling pathways. Cenicriviroc (CVC) aims to prevent recruitment of monocytes, macrophages, lymphocytes, and hepatic stellate cells via dual CCR2/CCR5 inhibition. CVC has previously demonstrated improved inflammatory and fibrosis makers in a rat thioacetamide-induced liver fibrosis model and a diet induced NASH mouse model (154). Though this are not cholestatic models of liver disease, the improvement in fibrosis may still be relevant and current studies are ongoing in adults with liver disease (NCT02217475). If effective, these agents can be studied in pediatric cholestatic diseases with significant hepatic fibrosis, particularly biliary atresia.

## Hepatocyte Transplant, Stem Cell Infusions, and Gene Therapies

Hepatocytes that could repopulate the liver with fully functional cells would be helpful not only in pediatric cholestatic liver diseases but also in several non-cholestatic and adult liver diseases. In one study specifically related to pediatric cholestatic liver disease, splenic injection of mdr2^−/−^ mice with MDR3-expressing hepatocytes while on a standard diet lead to modest improvement in phospholipid excretion in mice, but did lead to histologic improvement of disease and appeared to decrease the development of hepatic tumors (155). A more recent study with the mdr2^−/−^ mouse infused via the portal vein with mdr2^+/+^ hepatocytes had improved engraftment rates with concomitantly given glyceryl trinitrate (a vasodilator) (156). Prior work with BSEP knout out mice (a model of human PFIC 2) had also demonstrated favorable outcomes after wild-type hepatocyte transplantation (157). However, in most humans with cholestatic diseases treated with hepatocyte transplantation the effects have been less encouraging than in animal studies. A review nearly 15 years ago reported the use of infused hepatocytes in pediatric patients with PFIC 2 without improvement in disease, though apparently no significant complications (158). This study also summarized outcomes in several other liver-based metabolic diseases (though not primarily cholestatic diseases) with hepatocyte transplantation which had variable rates of success and may be of interest to the reader (158). A follow-up study to this one which reviewed outcomes in these same patients that progressed to need liver transplantation months after hepatocyte transplant demonstrated no evidence of engraftment of donor hepatocytes into liver cell plates on explanted livers (159). Though advances have been made over the years, the field of human hepatocyte transplantation as a therapy for liver diseases is still working to optimize cell quality/storage, increase engraftment rates, and allow for long term monitoring of these cells. A recent review of the current status of human hepatocyte transplantation for liver diseases and cirrhosis can be found elsewhere (160, 161).

Stem cell infusions for patients with liver disease has been proposed to alter hepatic inflammation and fibrosis which makes it a possible favorable treatment for pediatric cholestatic liver diseases. Additionally, if autologous stem cells are used then there would not be a requirement for immunosuppression (unlike hepatocyte transplantation). In a mouse model of biliary atresia, animals were given bone marrow-derived mesenchymal stem cells via intraperitoneal injection and the authors reported significantly improved AST, ALT, total and direct bilirubin along with improved histologic features and markers of hepatic fibrosis 14 days later (162). There is a case report of human hepatic progenitor cells being infused through the hepatic artery for a 1 year old patient with reported biliary atresia which reported improvement in several lab values including bilirubin at 2 months post-infusion (163). They reported they had followed the patient for at least 6 months after infusion, however no further laboratory values after 2 months post infusion or long-term patient outcome was reported in this case report. A study out of India of 26 patients with biliary atresia gave 11 patients an infusion of autologous mononuclear bone marrow stem cell infusion via the hepatic artery or portal vein at the time of their operative evaluation +/− Kasai surgery for biliary atresia (2/11 patients did not receive Kasai surgery due to severity of portal hypertension at time of diagnosis) while the remaining 15 patients received standard care (2/15 patients did not receive Kasai due to severity of portal hypertension at time of diagnosis) and then followed patients for at least 1 year or until death (164). The authors report a significant decrease in post-operative serum bilirubin with the use of stem cell infusion compared to the group without stem cell infusion at 7 d post-operatively and 6 months post-operatively. However, it is notable that in the cohort of patients that did not receive the stem cell infusions there were significantly more episodes of cholangitis which may accelerate the hepatic decline in biliary atresia and may be a confounding factor in this study, and also there was a high mortality rate in this series and no significant difference in median post-operative survival time, though the authors proposed the stem cell infusions may have prolonged life in early infancy. There is also currently an open-label trial (NCT03468699) enrolling patients 1–15 years old with cirrhosis secondary biliary atresia after undergoing Kasai portoenterostomy where treatment will include 2 administrations of autologous bone marrow mononuclear cells infused via the hepatic artery. Excellent reviews of the use of variable stem cell therapies for a variety of pediatric and adult liver diseases are available elsewhere (165, 166).

Given the prevalence of diseases with known, single-gene mutations within the field of chronic pediatric cholestasis, gene-editing technologies are an attractive future therapeutic option. There have been human subjects treated with CRISPR edited T cells and hematopoietic stem and progenitor cells and clinical trials are ongoing (NCT03399448, NCT03745287). While there are no current studies underway with pediatric cholestasis, CRISPR/Cas-9 gene-editing or and other prime editing tools may offer promise for future gene editing therapies for patients with single gene or other known mutations.

## Conclusion

Chronic cholestatic liver diseases are a significant cause of morbidity and mortality within the pediatric population and there is a notable lack of specific medical therapies that improve outcomes in these patients. However, the past few decades a new understanding of the intricate signaling pathways involved in bile acid metabolism and transport has led to novel targets for treating these diseases in pediatric patients. These include strategies to limit cytotoxicity by changing the bile pool hydrophobicity, enhancing protein folding, altering the expression of bile acid and other liver and ileal transporters to promote choleresis, enhancing hepatic detoxifying enzymes, disrupting the enterohepatic circulation of bile acids and decreasing the production of bile acids. Other strategies can target signaling pathways to decrease inflammation and fibrosis in liver diseases, and potential future gene editing therapies may be promising for identified gene defects. Based on our ever increasing and more nuanced understanding of the underlying etiologies of specific patient genotypes and phenotypes with pediatric cholestasis, combination therapies tailored to specific patients may be employed in the future. However, additional studies are warranted to elucidate potential therapeutic agents in humans that are effective, and also have favorable tolerability and low risk of long-term side effects.

## Author Contributions

AK and RG contributed to this manuscript, to the concept of the work, to the drafting and revisions of the manuscript, gave final approval of the manuscript, and are in agreement to be accountable for all aspects of the work.

## Conflict of Interest

The authors declare that the research was conducted in the absence of any commercial or financial relationships that could be construed as a potential conflict of interest.
